# Active surveillance for vancomycin-resistant enterococci colonization in intensive care unit

**DOI:** 10.1186/1753-6561-5-S6-P23

**Published:** 2011-06-29

**Authors:** F Qiao, Y Xie

**Affiliations:** 1 Infection Control Department, Chengdu, China; 2 West China Hospital,Sichuan University, Chengdu, China

## Introduction / objectives

### Introduction

Asymptomatic vancomycin-resistant enterococci (VRE) colonization is known to precede actual infection. The VRE isolation rate has rapidly increased in tertiary hospitals, but there is few data in this area of China, hindering the local infection control efforts.

### Objectives

To understand the local prevalence of VRE in patients hospitalized in ICU.

## Methods

A prospective observational study to estimate the prevalence of VRE colonization among inpatients in a 50-bed medical ICU was performed. From November 2010 to February 2011, rectal swabs were collected from inpatients at the time of ICU admission, a week later and at discharge from ICU and were then subject to culture and detection of VRE.

## Results

During the study period, 21(7.1%) out of 295 patients have been colonized with VRE at the time of ICU admission. Among the 295 patients included, 35 had a ICU stay less than 48 hours and their rectal swabs were not collected at discharge from ICU. A total of 133 patients were discharged from ICU within a week but beyond 48 h, among which 12 (9.0%) were colonized with VRE at discharge. The remaining127 patients were hospitalized in ICU for more than one week and 21 (16.5%) of them were positive for VRE at one-week ICU stay and 24 (18.9%) were positive at discharge.

## Conclusion

Although infection due to VRE is not common in China, the VRE colonization rate was not negligible among local medical ICU patients, especially when they had a ICU stay beyond one week. Activie survellaince is required to investigate the epidemiology of VRE in local settings.

## Disclosure of interest

None declared.

